# Role of proteoglycan synthesis genes in osteosarcoma stem cells

**DOI:** 10.3389/fonc.2024.1325794

**Published:** 2024-04-16

**Authors:** Ryoma Osumi, Kengo Sugihara, Makoto Yoshimoto, Kazuya Tokumura, Yuki Tanaka, Eiichi Hinoi

**Affiliations:** ^1^ Department of Bioactive Molecules, Pharmacology, Gifu Pharmaceutical University, Gifu, Japan; ^2^ United Graduate School of Drug Discovery and Medical Information Sciences, Gifu University, Gifu, Japan; ^3^ Center for One Medicine Innovative Translational Research, Division of Innovative Modality Development, Gifu University, Gifu, Japan

**Keywords:** osteosarcoma, osteosarcoma stem cell, proteoglycan, glycosaminoglycan, *β-1*, *3glucuronyltransferase 3*

## Abstract

Osteosarcoma stem cells (OSCs) contribute to the pathogenesis of osteosarcoma (OS), which is the most common malignant primary bone tumor. The significance and underlying mechanisms of action of proteoglycans (PGs) and glycosaminoglycans (GAGs) in OSC phenotypes and OS malignancy are largely unknown. This study aimed to investigate the role of PG/GAG biosynthesis and the corresponding candidate genes in OSCs and poor clinical outcomes in OS using scRNA-seq and bulk RNA-seq datasets of clinical OS specimens, accompanied by biological validation by *in vitro* genetic and pharmacological analyses. The expression of *β-1,3-glucuronyltransferase 3* (*B3GAT3*), one of the genes responsible for the biosynthesis of the common core tetrasaccharide linker region of PGs, was significantly upregulated in both OSC populations and OS tissues and was associated with poor survival in patients with OS with high stem cell properties. Moreover, the genetic inactivation of *B3GAT3* by RNA interference and pharmacological inhibition of PG biosynthesis abrogated the self-renewal potential of OSCs. Collectively, these findings suggest a pivotal role for *B3GAT3* and PG/GAG biosynthesis in the regulation of OSC phenotypes and OS malignancy, thereby providing a potential target for OSC-directed therapy.

## 1 Introduction

Osteosarcoma (OS) is the most common primary malignant bone tumor with a high risk of bone and lung metastases (1–4). The incidence of OS shows a bimodal age distribution, peaking in adolescents and young adults, and adults older than 65 years, and is slightly more common in men than in women (5, 6). OS is characterized by marked malignancy, strong invasiveness, rapid disease progression, and a high mortality rate (7, 8). OS commonly occurs in the knee joint (the metaphysis of the long tubular bones: the distal femur and the proximal tibia) (9, 10). The 5-year survival rate of OS stands at approximately 70% in the absence of metastases and decreases to 30% in patients with metastatic disease (11, 12). The exact cell origin of OS remains to be defined; however, it is believed to be cells of the osteoblast lineage, ranging from mesenchymal stem cells (MSCs) to osteoblast progenitors (13, 14). Osteosarcoma stem cells (OSCs) are functionally delineated based on their intrinsic properties, including self-renewal potential and multilineage differentiation capacity (15). OSCs also play a pivotal role in tumor initiation, recurrence, metastasis, and chemoresistance (16). Accumulating evidence suggests that targeting OSCs is an efficacious strategy for improving OS treatment (17, 18). Therefore, understanding the underlying molecular mechanisms governing the function of OSCs is necessary for developing novel therapeutic strategies for OS.

All mammalian glycosaminoglycans (GAGs), except hyaluronan (HA), attach to core proteins to form proteoglycans (PGs) (19–21). PGs/GAGs are abundantly distributed on the cell surface and in the extracellular matrix (22). GAGs have various biological functions and play important roles in numerous physiological and pathological conditions (23–25). Among them, the biosynthesis of chondroitin sulfate (CS); dermatan sulfate (DS), which is derived from CS by C5-epimerization of the β-D-glucuronic acid residue; and heparan sulfate (HS) begins with the formation of a common tetrasaccharide linker region to the core protein, followed by repeated addition of disaccharide units (26–28). The biosynthesis of the tetrasaccharide linker region in CS, DS, and HS is initiated by the enzymatic transfer of xylose to specific serine residues located in the core proteins of PGs within the endoplasmic reticulum by xylosyltransferase-I (XylT-I) and -II (XylT-II), encoded by *xylosyltransferase 1* (*XYLT1*) and *XYLT*2, respectively (29–31). Subsequently, two galactoses and a glucuronic acid are successively added to the xylose residues within the Golgi apparatus through the concerted actions of galactosyltransferase-I (GalT-I), galactosyltransferase-II (GalT-II), and glucuronyltransferase-I (GlcAT-I), which are encoded by *β-1,4-galactosyltransferase 7* (*B4GALT7*), *β-1,3-galactosyltransferase 6* (*B3GALT6*), and *β-1,3-glucuronyltransferase 3* (*B3GAT3*), respectively (32, 33).

PGs/GAGs not only play fundamental and diverse roles in the progression, malignancy, metastasis, and refractoriness of various types of cancer, but are also implicated in the cellular properties of cancer stem cells (CSCs) in some cancers, including glioblastoma, triple-negative breast cancer, and colorectal cancer (34, 35). Although some studies have been conducted to understand the role of PGs/GAGs in the pathogenesis of OS, limited data are available on the significance of enzymes related to PG/GAG biosynthesis in OSCs and OS malignancy. This study aimed to investigate the role of PG/GAG biosynthesis and corresponding candidate genes in OSCs and poor clinical outcomes in OS by combining bioinformatics analysis of clinical OS specimens with independent cohorts and *in vitro* genetic and pharmacological analyses.

## 2 Materials and methods

### 2.1 scRNA-seq data analysis

We analyzed two scRNA-seq datasets (GSE152048 and GSE162454) (36, 37). The GSE152048 dataset included 11 patients (five men and six women, 11–38 years). The data of five patients with primary osteoblastic OS lesions were used in subsequent analyses. The GSE162454 dataset included six primary OS patients (four men and two women, 13–45 years). The data of all six patients were used in subsequent analyses.

**Figure 1:**
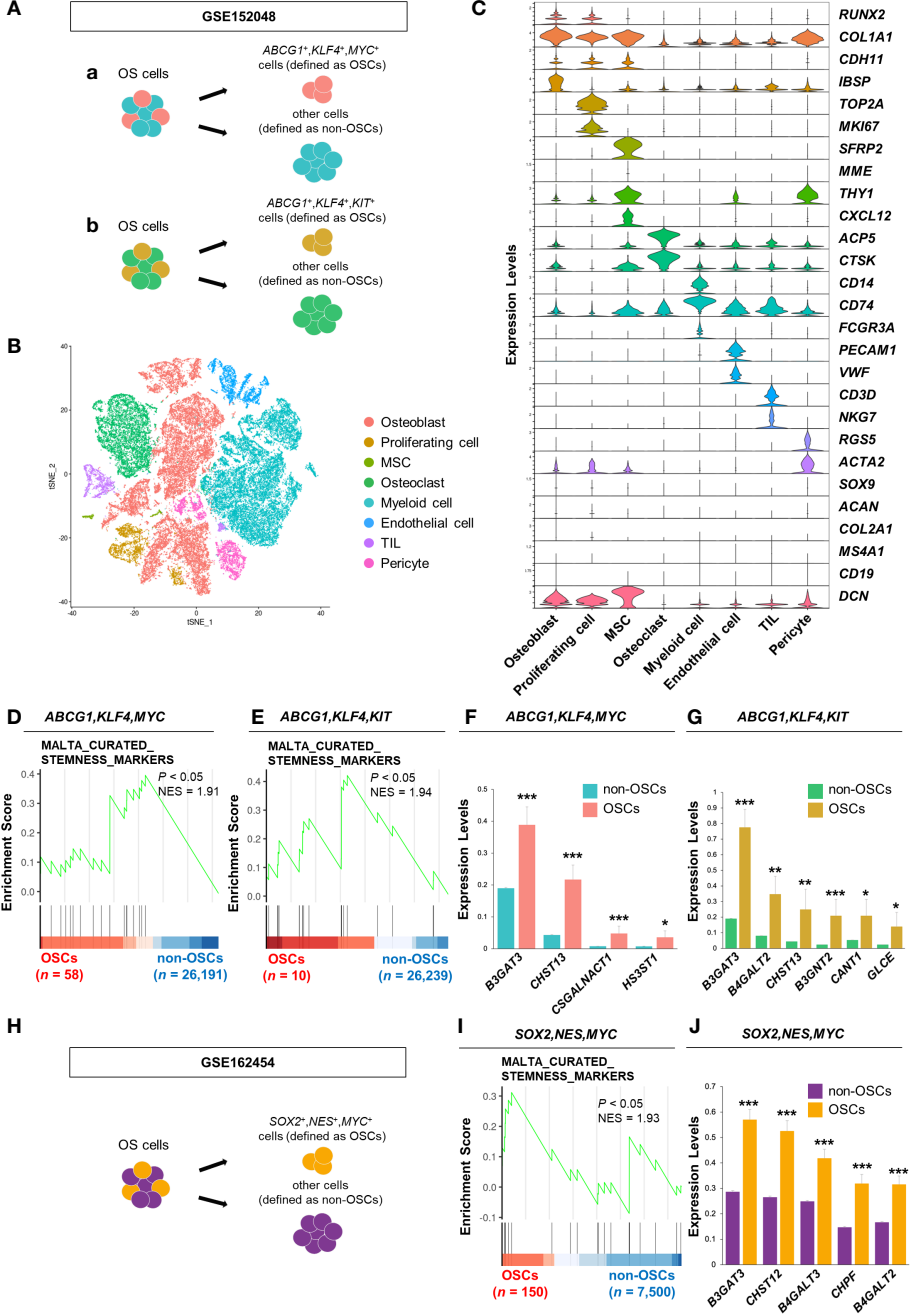
*B3GAT3* is upregulated in the OSC population of patients with OS. **(A)** Schematic of the identification of the OSC population in GSE152048. (a) *ABCG1*, *KLF4*, and *MYC* co-expressing cells or (b) *ABCG1*, *KLF4*, and *KIT* co-expressing cells were defined as OSCs, respectively. **(B)** t-SNE plot of cell clusters classified in OS tissues. **(C)** Violin plots showing the normalized expression levels of 27 representative marker genes across 8 clusters. **(D, E)** Enrichment plot for a gene set related to “stemness” between OSCs ([D] *ABCG1*, *KLF4*, and *MYC* or [E] *ABCG1*, *KLF4*, and *KIT* co-expressing cells) and non-OSCs. **(F, G)** Barplot showing the expression levels of PG/GAG biosynthesis genes between OSCs ([F] *ABCG1*, *KLF4*, and *MYC* or [G] *ABCG1*, *KLF4*, and *KIT* co-expressing cells) and non-OSCs. (*P < 0.05, **P < 0.01, ***P < 0.001). **(H)** Schematic of the identification of the OSC population in GSE162454. *SOX2*, *NES*, and *MYC* co-expressing cells were defined as OSCs. **(I)** Enrichment plot for a gene set related to “stemness” between OSCs and non-OSCs. **(J)** Barplot showing the expression levels of PG/GAG biosynthesis genes between OSCs and non-OSCs. The top five most highly expressed genes in OSCs are shown (***P < 0.001).

Data were analyzed using the “Seurat” package (version 4.3.0.1) in R (version 4.3.0) (38–40). First, the data were read using the Read10X function. In the preprocessing of each dataset, cells with > 6,000 and < 300 expressed genes with more than 10% mitochondrial RNA counts were considered low-quality and filtered out. The gene expression levels of the remained cells were normalized by regressing mitochondrial mapping rates on glmGamPoi using the SCTransform function. To remove batch effects, integration of the five sample datasets in GSE152048, and the six sample datasets in GSE152048, was performed using the SelectIntegrationFeatures, PrepSCTIntegration, RunPCA, FindIntegrationAnchors, and IntegrateData functions. Accordingly, 59,738 cells in GSE152048 and 32,681 cells in GSE162454 were used for downstream analysis, respectively. For dimensional reduction, principal component analysis (PCA) and t-distributed stochastic neighbor embedding (t-SNE) were performed using the RunPCA and RunTSNE functions. To cluster cell populations, k.param nearest neighbors were calculated using the FindNeighbors function using the first 50 principal components. Clusters were identified using theFindClusters function at a resolution of 0.2. Each cluster was manually annotated based on violin plots of the expression of established cell-specific marker genes. Detailed information on these marker genes is provided in **Figure 1C** and **Supplementary Figure 1B**. Osteoblasts, proliferating cells, and MSCs were extracted as OS cells from the identified clusters (*n* = 26,249 in GSE152048, *n* = 7,650 in GSE162454).

OS cells were divided into two groups, OSCs and non-OSCs, for downstream analysis. In GSE152048, *ABCG1*, *KLF4*, and *MYC* co-expressing cells were defined as OSCs (*n* = 58) and others as non-OSCs (*n* = 26,191). Similarly, *ABCG1*, *KLF4*, and *MYC* co-expressing cells were defined as OSCs (*n* = 10) and others as non-OSCs (*n* = 26,239). In GSE162454, *SOX2*, *NES*, and *MYC* co-expressing cells were defined as OSCs (*n* = 150) and others as non-OSCs (*n* = 7,500).

Sixty-three human PG/GAG biosynthesis-related genes were obtained by integrating four gene sets (KEGG_GLYCOSAMINOGLYCAN_BIOSYNTHESIS_CHONDROITIN_SULFATE, KEGG_GLYCOSAMINOGLYCAN_BIOSYNTHESIS_HEPARAN_SULFATE, KEGG_GLYCOSAMINOGLYCAN_BIOSYNTHESIS_KERATAN_SULFATE, and WP_PROTEOGLYCAN_BIOSYNTHESIS) registered in the MSigDB database (http://gsea-msigdb.org/gsea/msigdb/index.jsp). Differentially expressed genes (DEGs) were identified among these 63 genes using Wilcoxon’s rank-sum test (*P* < 0.05) using the wilcoxauc function in the “presto” package (version 1.0.0). Gene Set Enrichment Analysis (GSEA) was performed using the GSEA function (minGSsize, 5; maxGSsize, 500; eps, 0; pvalueCutoff, 0.05) in the “clusterProfiler” package (version 4.8.3). The gene sets used in GSEA were obtained from the C2 and C5 collections in the MSigDB database using the msigdbr function in the “msigdbr” package (version 7.5.1). Gene sets with NES > 0 and *P* < 0.05 were considered significantly enriched. Visualization was performed using the gseaplot2 function in the “enrichplot” package (version 1.20.1).

### 2.2 Bulk RNA-seq data analysis

We analyzed the RNA-seq dataset (PRJNA539828) obtained from OS (*n =* 16) and non-tumor (*n =* 4) tissues from patients with OS (41). Fastq files were downloaded using “SRA Toolkit” (version 3.0.1). Trimming was performed using “Trim_Galore” (version 0.6.7). Quality control after trimming was performed using “FASTQC” (version 0.12.1). Mapping to the hg38 human genome assembly was performed using “STAR” (version 2.7.10b). Expression levels were calculated from the bam files generated by mapping using “RSEM” (version 1.3.3). GSEA was performed using the “clusterProfiler” package (version 4.8.3) in R (version 4.3.0). Visualization of the GSEA results was performed using the “enrichplot” package (version 1.20.3).

### 2.3 Survival analysis

Clinical data from patients with OS were downloaded from the TARGET-OS database. Patients were divided into high and low expression groups based on median gene expression values. Survival analysis was conducted with the log-rank test using the “survival” package (version 4.8.3). Kaplan–Meier curves were plotted using the “survminer” package (version 0.4.9).

### 2.4 Cell culture

HEK293T cells were obtained from the RIKEN Cell Bank (Saitama, Japan) and cultured in DMEM (FUJIFILM Wako Pure Chemical) supplemented with 10% FBS (Hyclone) and 1% penicillin/streptomycin (Thermo Fisher Scientific) at 37°C in 5% CO_2_ (42). The patient-derived OS cell line 143B was obtained from the ATCC (Manassas, USA) and cultured in adherent medium containing DMEM supplemented with 10% FBS, 110 μg/mL sodium pyruvate (FUJIFILM Wako Pure Chemical), and 1% penicillin/streptomycin. Both cell types were cultured in tissue culture dishes (SARSTEDT) to ensure optimal adherence and expansion. To enrich stem-like cells, 143B cells were harvested using trypsin (BD Bioscience) and EDTA (FUJIFILM Wako Pure Chemical), then cultured in osteosphere medium containing DMEM/F12 (FUJIFILM Wako Pure Chemical) supplemented with 20 ng/mL recombinant human EGF (FUJIFILM Wako Pure Chemical), 20 ng/mL recombinant human basic FGF (FUJIFILM Wako Pure Chemical), B27 supplement without vitamin A (Gibco), GlutaMAX (Thermo Fisher Scientific), and 1% penicillin/streptomycin. Under these conditions, the cells were incubated in Ultra-Low Attachment Surface culture dishes (Corning). To assess the differentiation potential of OSCs, the cells were transferred from osteosphere to adherent medium, and from Ultra-Low Attachment Surface to tissue culture dishes, to promote adherence and differentiation.

### 2.5 Lentiviral transfection

To introduce vectors into HEK293T cells, the calcium phosphate method was employed (43). Lentiviral vectors containing expression constructs, pRRE and pREV packaging plasmids, and VSVG envelope plasmids were transfected into HEK293T cells for packaging. After 48 h of transfection, viral supernatants were harvested and subsequently incubated with 143B cells for 24 h. Following this, 143B cells were selected by culturing them for 4 days in the presence of 0.5 μg/mL puromycin prior to their use in experiments. Plasmid pLKO.1-sh*B3GAT3* (TRCN0000035610) was purchased from Sigma-Aldrich; pLKO.1 puro plasmid (#8453) was purchased from Addgene.

### 2.6 Sphere formation and limiting dilution assay

For sphere formation assay, 143B cells (1,000 cells) were seeded in ultra-low attachment 96-well plates (Corning) and cultured in osteosphere medium supplemented with 1% methylcellulose (FUJIFILM Wako Pure Chemical). The number of spheres was calculated on the fifth day using a BZ-X800 microscope (KEYENCE). Sphere formation ability was assessed by enumerating the quantity of spheres with a diameter > 30 μm (44). For limiting dilution assay, cells were seeded in 96-well plates at a density of 1, 5, 10, 20, 40, or 80 cells/well with five replicates per density. The presence of spheres in each well was determined after 5 days. Wells containing spheres with a diameter > 50 μm were considered positive, while those without spheres were considered negative. The frequency of sphere formation was assessed using an extreme limiting dilution algorithm (ELDA software; http://bioinf.wehi.edu.au/software/elda/).

### 2.7 Reverse transcription quantitative PCR

Total cellular RNA was isolated. cDNA was synthesized using reverse transcriptase and oligo-dT primers (45). RT-qPCR analysis was performed using gene-specific primers and THUNDERBIRD SYBR qPCR Mix (TOYOBO) on an MX3000P instrument (Agilent Technologies). mRNA expression levels were standardized using *GAPDH* as an internal control (46). The primer sequences used in this study are listed in the **Supplementary Table**.

### 2.8 Flow cytometry

143B cells (1,000,000 cells) were incubated with Fixable Viability Stain 780 (1:1000, #565388, BD) for 10 minutes at room temperature in the dark, followed by incubation with APC-CD133 (1:50, #566597, BD) for 30 minutes at 4°C in the dark. Samples were analyzed using a CytoFLEX S (Beckman).

### 2.9 Xenograft model of OS

Animal experiments were performed in accordance with the Guidelines for the Care and Use of Laboratory Animals of Gifu Pharmaceutical University. Four-week-old female BALB/c nu/nu mice were obtained from Japan SLC (Hamamatsu, Japan). Mice were injected subcutaneously with 5 × 10^6^ 143B cells. Tumor length and width were measured with calipers. Tumor volume was calculated as (length × width^2^)/2. Mice were euthanized before the tumor length exceeded 20 mm.

### 2.10 Statistical analysis

Unless otherwise specified, data are expressed as mean ± SE. Statistical significance was assessed using Student’s *t*-test. A *P <* 0.05 was considered statistically significant.

## 3 Results

### 3.1 *B3GAT3* is upregulated in the OSC population of OS patient specimens

First, we analyzed a scRNA-seq dataset of clinical OS specimens deposited in the GEO database (GSE152048), to profile the properties of OSCs (**Figure 1A**). Eight clusters were identified through t-SNE analysis based on the genetic profiles of the cells (**Figure 1B**). Canonical markers were used to annotate the different cell types: osteoblasts (*RUNX2* ^+^ *,COL1A1* ^+^ *,CDH11* ^+^ *,IBSP* ^+^), proliferating cells (*TOP2A* ^+^ *,MKI67* ^+^), MSCs (*SFRP2* ^+^ *,MME* ^+^ *,THY1* ^+^ *,CXCL12* ^+^), osteoclasts (*ACP5* ^+^ *,CTSK* ^+^), myeloid cells (*CD14* ^+^ *,CD74* ^+^ *,FCGR3A* ^+^), endothelial cells (*PECAM1* ^+^ *,VWF* ^+^), tumor infiltrating lymphocytes (TILs; *CD3D* ^+^ *,NKG7* ^+^), and pericytes (*RGS5* ^+^ *,ACTA2* ^+^) (**Figure 1C**). Malignant cells were distinguished from non-malignant cells using CNV inference (data not shown). The OS cell population was further divided into two groups, OSCs and non-OSCs, based on the co-expression of three stem cell markers, *ABCG1*, *KLF4*, and *MYC* (**Figure 1A**a). The enrichment of the gene set involved in “stemness” in OSCs was confirmed by GSEA (**Figure 1D**). Consistent results were obtained when another cell population with co-expression of stem cell markers (*ABCG1*, *KLF4*, and *KIT*) was defined as OSCs (**Figures 1A**b, **E**), allowing us to define these cells as the OSC population. Under these experimental conditions, we identified DEGs related to the biosynthesis of PGs/GAGs between OSCs and non-OSCs. Sixty-three DEGs related to the biosynthesis of PGs/GAGs were screened, with four and six significantly upregulated genes in OSCs defined by co-expression of *ABCG1*/*KLF4*/*MYC*, and *ABCG1*/*KLF4*/*KIT*, respectively (**Figures 1F, G**). Among the significantly upregulated genes, *B3GAT3*, one of the genes responsible for the biosynthesis of the common core tetrasaccharide linker region of PGs (47), was the most highly expressed gene in both *ABCG1*/*KLF4*/*MYC* and *ABCG1*/*KLF4*/*KIT* OSC populations (**Figures 1F, G**).

To confirm the results obtained from GSE152048, we analyzed a different scRNA-seq dataset (GSE162454) (**Supplementary Figures 1A, B**). The OS cell population was divided into two groups, OSCs and non-OSCs, based on the co-expression of three stem cell markers, *SOX2*, *NES*, and *MYC* (**Figure 1H**). The enrichment of the gene set involved in “stemness” in OSCs was confirmed by GSEA (**Figure 1I**). Among the 63 genes related to the biosynthesis of PGs/GAGs, *B3GAT3* was significantly upregulated in OSCs defined by co-expression of *SOX2*/*NES*/*MYC* (**Figure 1J**, **Supplementary Figure 1C**), with consistent results from two independent cohorts of clinical specimens.

### 3.2 *B3GAT3* is associated with poor prognosis in patients with OS with high stem cell properties

**Figure 2:**
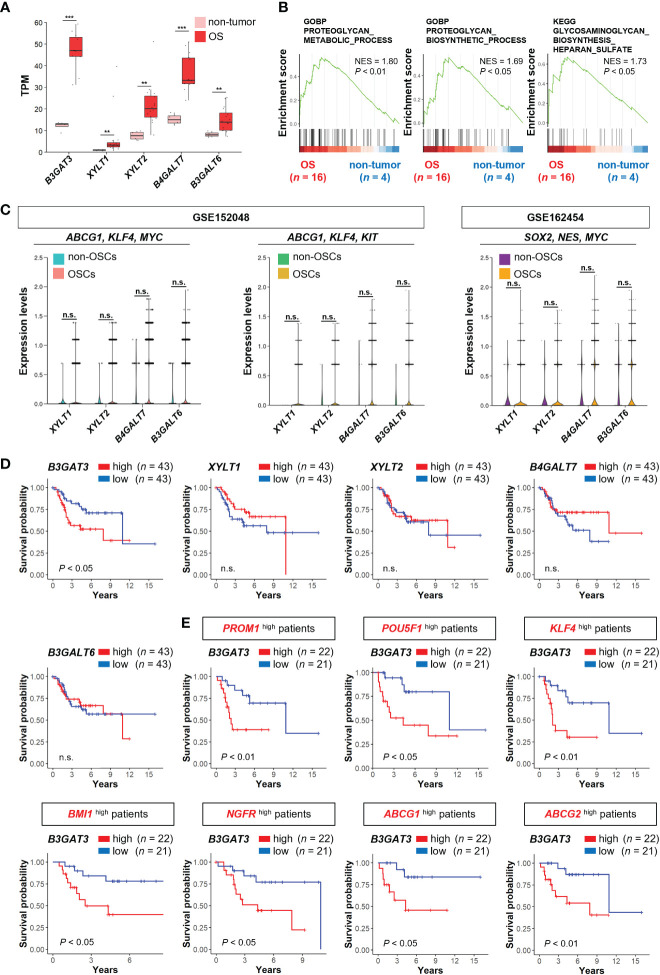
*B3GAT3* is associated with poor prognosis in OS patients with high stemness. **(A)** The expression levels of *B3GAT3*, *XYLT1, XYLT2, B4GALT7*, and *B3GALT6* in OS (*n* = 16) and non-tumor (*n* = 4) tissues using bulk RNA-seq dataset (PRJNA539828) (**P < 0.01, ***P < 0.001). **(B)** The enrichment plots for gene sets related to “PG metabolic process”, “PG biosynthetic process”, and “GAG biosynthesis” in OS (*n* = 16) and non-tumor (*n* = 4) tissues. **(C)** The expression levels of *XYLT1, XYLT2, B4GALT7*, and *B3GALT6* in OSCs and non-OSCs using scRNA-seq datasets (GSE152048 and GSE162454). **(D)** Kaplan–Meier curves comparing patients with OS with high (*n* = 43) and low (*n* = 43) expression levels of *B3GAT3*, *XYLT1*, *XYLT2*, *B4GALT7*, and *B3GALT6* respectively. **(E)** Kaplan–Meier curves comparing high (*n* = 22) and low (*n* = 21) *B3GAT3* expression levels in patients with OS with high stemness. n.s., not significant.

Next, we determined the expression levels of genes related to the biosynthesis of the common core tetrasaccharide linker region of PGs in clinical OS tissues. The expression levels of *B3GAT3*, *XYLT1, XYLT2, B4GALT7*, and *B3GALT6* were significantly upregulated in OS tissues compared to those in non-tumor tissues, according to the analysis of the bulk RNA-seq dataset (PRJNA539828) (**Figure 2A**). GSEA revealed significant enrichment of gene sets related to the “PG metabolic process”, “PG biosynthetic process”, and “GAG biosynthesis (HS)” (**Figure 2B**), which contain the above five PG biosynthesis genes. Contrary to the significant upregulation of *B3GAT3* in OSC populations (**Figures 1F, G, J**), the expression levels of *XYLT1, XYLT2, B4GALT7*, and *B3GALT6* did not differ significantly between OSCs and non-OSCs, even when OSC populations were defined under three different conditions (**Figure 2C**).

Next, we assessed whether the expression levels of *B3GAT3*, *XYLT1, XYLT2, B4GALT7*, and *B3GALT6* affected the survival of patients with OS using the TARGET-OS database. Kaplan–Meier analysis revealed that patients with OS with higher *B3GAT3* expression had significantly shorter survival than those with lower *B3GAT3* expression (**Figure 2D**). In contrast, the expression levels of *XYLT1*, *XYLT2*, *B4GALT7*, and *B3GALT6* were not significantly correlated with the prognosis of patients with OS (**Figure 2D**). Given that only *B3GAT3* expression was correlated with poor prognosis in patients with OS, we next assessed whether *B3GAT3* expression was associated with poor prognosis in patients with OS harboring higher stem cell properties. Kaplan–Meier analysis demonstrated that high *B3GAT3* expression was significantly associated with poor prognosis in patients with high expression of stemness markers, such as *PROM1*, *POU5F1*, *KLF4*, *BMI1*, *NGFR*, *ABCG1*, and *ABCG2* (**Figure 2E**).

### 3.3 Targeting *B3GAT3* impairs the self-renewal potential of 143B OS cells *in vitro*

**Figure 3:**
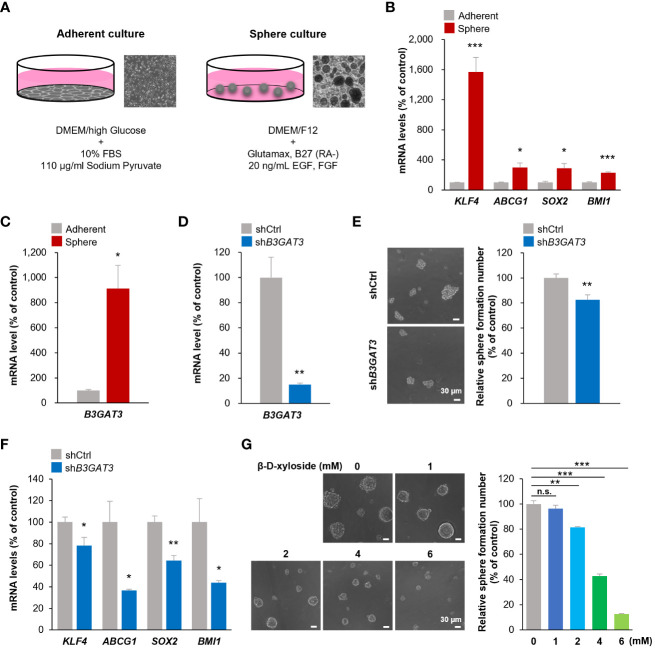
Inhibition of *B3GAT3* suppresses the self-renewal ability of 143B OS cells *in vitro*. **(A)** 143B cells were cultured under sphere or adherent conditions. **(B)** The mRNA expression levels of *KLF4*, *ABCG1*, *SOX2*, and *BMI1* were determined in sphere and adherent cells using RT-qPCR (*n* = 4. *P < 0.05, ***P < 0.001). **(C)** The mRNA expression level of *B3GAT3* was determined in sphere and adherent cells using RT-qPCR (*n* = 4. *P < 0.05). **(D)** *B3GAT3* knockdown was verified via RT-qPCR (*n* = 5. **P < 0.01). **(E)** The sphere formation ability of 143B cells was assessed following *B3GAT3* knockdown. Representative images are presented (left, scale bar = 30 μm). The number of spheres was counted (right, *n* = 8. **P < 0.01). **(F)** The mRNA expression levels of *KLF4*, *ABCG1*, *SOX2*, and *BMI1* were determined in *B3GAT3* knockdown 143B cells (*n* = 4. *P < 0.05, **P < 0.01). **(G)** 143B cells were treated with β-D-xyloside (0, 1, 2, 4, 6 mM), and sphere formation ability was assessed. Representative images are presented (left, scale bar = 30 μm). The number of spheres was counted (right, *n* = 5. **P < 0.01, ***P < 0.001 using Student’s *t*-test with Holm-Sidak correction for multiple comparisons). The mRNA expression level (normalized to *GAPDH*) is presented relative to that in **(B, C)** adherent cells and **(D, F)** cells treated with shCtrl. n.s., not significant.

To validate the results of the bioinformatics analysis, 143B cells were cultured under floating or adherent conditions, followed by determination of *B3GAT3* expression (**Figure 3A**). First, we confirmed the stemness and tumorigenicity of 143B cells *in vitro* and *in vivo* as previously demonstrated (48, 49). Under floating condition, 143B cells formed tumorspheres and exhibited self-renewal potential in the sphere formation and limited dilution assays, respectively (**Supplementary Figures 2A, B**), along with higher expression levels of the stem cell markers, *KLF4*, *ABCG1*, *SOX2*, and *BMI1* (**Figure 3B**). The proportion of CD133^+^ cells were markedly increased in 143B tumorspheres (**Supplementary Figure 2C**). 143B tumorspheres differentiated into 143B cells under adherent conditions (**Supplementary Figure 2D**). The tumorigenicity of 143B cells was confirmed in an orthotopic xenograft mouse model (**Supplementary Figure 2E**). Under these conditions, *B3GAT3* expression was significantly upregulated in 143B tumorspheres compared to differentiated 143B cells (**Figure 3C**).

Next, we elucidated the functional significance of GlcAT-I/*B3GAT3* in 143B cells *in vitro* by targeting *B3GAT3* expression using lentiviral shRNA. *B3GAT3* mRNA levels were markedly reduced by sh*B3GAT3* in 143B cells (**Figure 3D**). Disruption of *B3GAT3* with shRNA significantly decreased tumorsphere formation ability of 143B cells (**Figure 3E**). Furthermore, *B3GAT3* knockdown resulted in a significant downregulation of the stem cell markers, *KLF4*, *ABCG1*, *SOX2*, and *BMI1* in 143B tumorspheres (**Figure 3F**). Next, we determined whether the pharmacological inhibition of PG biosynthesis by 4-nitrophenyl β-D-xylopyranoside (β-D-xyloside), an inhibitor of GAG chain attachment to the core protein (50), could confirm the genetic inhibition of *B3GAT3* in 143B cells. β-D-xyloside significantly decreased the tumorsphere formation ability of 143B cells at concentrations > 2 mM in a concentration-dependent manner (**Figure 3G**). Although further studies should be performed to demonstrate the pivotal role of *B3GAT3* on OSC properties by purifying stem cells from 143B tumorspheres because of their heterogeneous population including a subset exhibiting OSC markers, these genetic and pharmacological analyses indicate that *B3GAT3* and PG/GAG biosynthesis could be implicated in the regulation of stem cell properties of 143B *in vitro*.

## 4 Discussion

PGs/GAGs are widely recognized as important regulators of stem cell function in embryonic development and tissue regeneration (51, 52). Moreover, the aberrant functions of PGs/GAGs have recently been shown to contribute to CSC phenotypes, tumor initiation, recurrence, metastasis, and chemoresistance (35). The assembly of HS, CS, and DS is initiated by the formation of a common tetrasaccharide structure (Xyl-Gal-Gal-GlcA), catalyzed by XylT-I, XylT-II, GalT-I, GalT-II, and GlcAT-I, encoded by *XYLT1*, *XYLT2*, *B4GALT7*, *B3GALT6*, and *B3GAT3*, respectively (26–28). Mutations in these genes can cause inherited diseases that result in various bone, skin, and connective tissue abnormalities (53, 54). For instance, mutations in *B3GAT3* have been implicated in multiple joint dislocations, short stature, and craniofacial dysmorphism, with or without congenital heart defects (47). However, the importance of PG/GAG biosynthesis and the functional roles of the corresponding genes (*XYLT1*, *XYLT2*, *B4GALT7*, *B3GALT6*, and *B3GAT3*) in OSC properties and OS pathogenesis are largely unknown. Although further *in vivo* analyses should be performed to validate our findings, to our knowledge, this is the first study to reveal, using integrated bioinformatics analysis and *in vitro* genetic and pharmacological studies, that the PG/GAG biosynthesis pathway and corresponding enzyme, GlcAT-I/*B3GAT3*, may be associated with the maintenance of OSC characteristics and OS malignancy.

Notably, the expression analysis of DEGs related to the biosynthesis of PGs/GAGs revealed the potential involvement of alternative candidate genes in OSC properties. Carbohydrate sulfotransferase 13 (*CHST13*), which catalyzes the transfer of sulfate to position 4 of the GalNAc residue of chondroitin (55), was the commonly significantly upregulated gene in all three OSC populations defined by the co-expression of *ABCG1*/*KLF4*/*MYC*, and *ABCG1*/*KLF4*/*KIT*, and *SOX2*/*NES*/*MYC* (**Figures 1F, G, J**, **Supplementary Figure 1C**). In addition to *B3GAT3* and *CHST13*, there were several significantly upregulated genes in each OSC population without overlap, indicating that these additional genes require further exploration. OS is highly heterogeneous in terms of molecular pathogenesis, which is at least in part due to the genetic and phenotypic variation in OSCs, suggesting that optimal biomarkers vary slightly between patients and cancer types (56, 57). For that reason, different OSC markers were used for each of the datasets: GSE152048 and GSE162454. It is also noteworthy that there were discrepancies in the expression of PG biosynthesis genes between OSC populations and OS tissues. Only *B3GAT3* was significantly upregulated in OSC populations (**Figures 1F, G, J**, **2C**). However, all five PG biosynthesis genes (*XYLT1*, *XYLT2*, *B4GALT7*, *B3GALT6*, and *B3GAT3*) were significantly upregulated in OS tissues (**Figure 2A**), in which the proportion of OSC is small. Therefore, it can be speculated that XylT-I/*XYLT1*, XylT-II/*XYLT2*, GalT-I/*B4GALT7*, and GalT-II/*B3GALT6* may have functional roles in differentiated OS cell properties rather than in OSC properties, providing an incentive to pursue further research to determine their roles in OS pathogenesis in cell culture studies.

The primary therapeutic approach for OS is a combination of surgical intervention and chemotherapy. Effective treatments for OS have not improved over the past four decades (3, 4). Although accumulating evidence suggests that mutations in the tumor suppressor genes, *RB1* and *TP53*, are associated with the development of OS, cytogenetic analysis suggests that genomic profiles differ significantly among patients with OS, without specific patterns, resulting in difficulties in the development of new and effective drugs and innovative treatment strategies (58–62). Our findings contribute to the improvement of our understanding of the molecular mechanisms underlying OS development and progression, as well as OSC properties, and suggest that PG/GAG biosynthesis and the corresponding genes expressed by OSCs may represent novel and effective targets for drug development to treat OS in humans.

## Data availability statement

The datasets presented in this study can be found in online repositories. The names of the repository/repositories and accession number(s) can be found in the article/**Supplementary Material**.

## Ethics statement

Ethical approval was not required for the studies on humans in accordance with the local legislation and institutional requirements because only commercially available established cell lines were used.

## Author contributions

EH: Writing – original draft, Writing – review & editing, Conceptualization, Funding acquisition, Supervision. RO: Writing – original draft, Writing – review & editing, Conceptualization, Formal analysis, Investigation, Visualization. KS: Writing – original draft, Writing – review & editing, Conceptualization, Formal analysis, Investigation, Visualization. MY: Writing – original draft, Writing – review & editing, Formal analysis, Investigation, Methodology, Validation, Visualization. KT: Writing – original draft, Writing – review & editing, Formal analysis, Investigation, Methodology, Validation, Visualization. YT: Writing – original draft, Formal analysis, Investigation, Methodology, Validation.

## References

[1] Panez-Toro I Muñoz-García J Vargas-Franco JW Renodon-Cornière A Heymann MF Lézot F. Advances in osteosarcoma. Curr osteoporosis Rep. (2023) 21:330–43. doi: 10.1007/s11914-023-00803-9 PMC10393907 37329384

[2] Rickel K Fang F Tao J. Molecular genetics of osteosarcoma. Bone. (2017) 102:69–79. doi: 10.1016/j.bone.2016.10.017 27760307 PMC5393957

[3] Beird HC Bielack SS Flanagan AM Gill J Heymann D Janeway KA. Osteosarcoma. Nat Rev Dis Primers. (2022) 8:77. doi: 10.1038/s41572-022-00409-y 36481668

[4] Gill J Gorlick R. Advancing therapy for osteosarcoma. Nat Rev Clin Oncol. (2021) 18:609–24. doi: 10.1038/s41571-021-00519-8 34131316

[5] Brown HK Schiavone K Gouin F Heymann MF Heymann D. Biology of bone sarcomas and new therapeutic developments. Calcified Tissue Int. (2018) 102:174–95. doi: 10.1007/s00223-017-0372-2 PMC5805807 29238848

[6] Xie D Wang Z Li J Guo DA Lu A Liang C. Targeted delivery of chemotherapeutic agents for osteosarcoma treatment. Front Oncol. (2022) 12:843345. doi: 10.3389/fonc.2022.843345 35311145 PMC8931218

[7] Berner K Johannesen TB Berner A Haugland HK Bjerkehagen B Bøhler PJ. Time-trends on incidence and survival in a nationwide and unselected cohort of patients with skeletal osteosarcoma. Acta Oncol (Stockholm Sweden). (2015) 54:25–33. doi: 10.3109/0284186x.2014.923934 PMC4364276 24957555

[8] Ji Z Shen J Lan Y Yi Q Liu H. Targeting signaling pathways in osteosarcoma: Mechanisms and clinical studies. MedComm. (2023) 4:e308. doi: 10.1002/mco2.308 37441462 PMC10333890

[9] Yang Z Li X Yang Y He Z Qu X Zhang Y. Long noncoding RNAs in the progression, metastasis, and prognosis of osteosarcoma. Cell Death Dis. (2016) 7:e2389. doi: 10.1038/cddis.2016.272 27685633 PMC5059871

[10] Pang H Wu T Peng Z Tan Q Peng X Zhan Z. Baicalin induces apoptosis and autophagy in human osteosarcoma cells by increasing ROS to inhibit PI3K/Akt/mTOR, ERK1/2 and β-catenin signaling pathways. J Bone Oncol. (2022) 33:100415. doi: 10.1016/j.jbo.2022.100415 35573641 PMC9091934

[11] Zhao X Wu Q Gong X Liu J Ma Y. Osteosarcoma: a review of current and future therapeutic approaches. Biomed Eng Online. (2021) 20:24. doi: 10.1186/s12938-021-00860-0 33653371 PMC7923306

[12] Kansara M Teng MW Smyth MJ Thomas DM. Translational biology of osteosarcoma. Nat Rev Cancer. (2014) 14:722–35. doi: 10.1038/nrc3838 25319867

[13] Mutsaers AJ Walkley CR. Cells of origin in osteosarcoma: mesenchymal stem cells or osteoblast committed cells? Bone. (2014) 62:56–63. doi: 10.1016/j.bone.2014.02.003 24530473

[14] Franceschini N Verbruggen B Tryfonidou MA Kruisselbrink AB Baelde H de Visser KE. Transformed canine and murine mesenchymal stem cells as a model for sarcoma with complex genomics. Cancers. (2021) 13:1126. doi: 10.3390/cancers13051126 33807947 PMC7961539

[15] Yan GN Lv YF Guo QN. Advances in osteosarcoma stem cell research and opportunities for novel therapeutic targets. Cancer Lett. (2016) 370:268–74. doi: 10.1016/j.canlet.2015.11.003 26571463

[16] Fujiwara S Kawamoto T Kawakami Y Koterazawa Y Hara H Takemori T. Acquisition of cancer stem cell properties in osteosarcoma cells by defined factors. Stem Cell Res Ther. (2020) 11:429. doi: 10.1186/s13287-020-01944-9 33008481 PMC7532109

[17] Abarrategi A Tornin J Martinez-Cruzado L Hamilton A Martinez-Campos E Rodrigo JP. Osteosarcoma: cells-of-origin, cancer stem cells, and targeted therapies. Stem Cells Int. (2016) 2016:3631764. doi: 10.1155/2016/3631764 27366153 PMC4913005

[18] Menéndez ST Gallego B Murillo D Rodríguez A Rodríguez R. Cancer stem cells as a source of drug resistance in bone sarcomas. J Clin Med. (2021) 10. doi: 10.3390/jcm10122621 PMC8232081 34198693

[19] Muir H. Proteoglycans of cartilage. J Clin Pathol. (1978) 31:67–81. doi: 10.1136/jcp.31.Suppl_12.67 PMC1347125 365895

[20] Kjellén L Lindahl U. Proteoglycans: structures and interactions. Annu Rev Biochem. (1991) 60:443–75. doi: 10.1146/annurev.bi.60.070191.002303 1883201

[21] Couchman JR. Transmembrane signaling proteoglycans. Annu Rev Cell Dev Biol. (2010) 26:89–114. doi: 10.1146/annurev-cellbio-100109-104126 20565253

[22] Esko JD Kimata K Lindahl U. Proteoglycans and sulfated glycosaminoglycans. In: Varki A Cummings RD Esko JD Freeze HH Stanley P Bertozzi CR, editors. Essentials of glycobiology. Cold Spring Harbor Laboratory Press Copyright © 2009, The Consortium of Glycobiology Editors, La Jolla, California, Cold Spring Harbor (NY (2009).

[23] Mizumoto S Yamada S Sugahara K. Molecular interactions between chondroitin-dermatan sulfate and growth factors/receptors/matrix proteins. Curr Opin Struct Biol. (2015) 34:35–42. doi: 10.1016/j.sbi.2015.06.004 26164146

[24] Miller GM Hsieh-Wilson LC. Sugar-dependent modulation of neuronal development, regeneration, and plasticity by chondroitin sulfate proteoglycans. Exp Neurol. (2015) 274:115–25. doi: 10.1016/j.expneurol.2015.08.015 PMC4679498 26315937

[25] Soares da Costa D Reis RL Pashkuleva I. Sulfation of glycosaminoglycans and its implications in human health and disorders. Annu Rev Biomed Eng. (2017) 19:1–26. doi: 10.1146/annurev-bioeng-071516-044610 28226217

[26] Kreuger J Kjellén L. Heparan sulfate biosynthesis: regulation and variability. J Histochem cytochemistry: Off J Histochem Soc. (2012) 60:898–907. doi: 10.1369/0022155412464972 PMC3527889 23042481

[27] Mikami T Kitagawa H. Biosynthesis and function of chondroitin sulfate. Biochim Biophys Acta. (2013) 1830:4719–33. doi: 10.1016/j.bbagen.2013.06.006 23774590

[28] Sugahara K Kitagawa H. Recent advances in the study of the biosynthesis and functions of sulfated glycosaminoglycans. Curr Opin Struct Biol. (2000) 10:518–27. doi: 10.1016/s0959-440x(00)00125-1 11042448

[29] Götting C Kuhn J Brinkmann T Kleesiek K. Xylosylation of alternatively spliced isoforms of Alzheimer APP by xylosyltransferase. J Protein Chem. (1998) 17:295–302. doi: 10.1023/a:1022549121672 9588955

[30] Hinsdale ME. Xylosyltransferase I, II (XYLT1,2). In: Taniguchi N Honke K Fukuda M Narimatsu H Yamaguchi Y Angata T, editors. Handbook of glycosyltransferases and related genes. Springer Japan, Tokyo (2014). p. 873–83.

[31] Briggs DC Hohenester E. Structural basis for the initiation of glycosaminoglycan biosynthesis by human xylosyltransferase 1. Structure (London England: 1993). (2018) 26:801–9.e3. doi: 10.1016/j.str.2018.03.014 29681470 PMC5992326

[32] Wen J Xiao J Rahdar M Choudhury BP Cui J Taylor GS. Xylose phosphorylation functions as a molecular switch to regulate proteoglycan biosynthesis. Proc Natl Acad Sci United States America. (2014) 111:15723–8. doi: 10.1073/pnas.1417993111 PMC4226115 25331875

[33] Koike T Izumikawa T Tamura J Kitagawa H. FAM20B is a kinase that phosphorylates xylose in the glycosaminoglycan-protein linkage region. Biochem J. (2009) 421:157–62. doi: 10.1042/bj20090474 19473117

[34] Ibrahim SA Hassan H Vilardo L Kumar SK Kumar AV Kelsch R. Syndecan-1 (CD138) modulates triple-negative breast cancer stem cell properties via regulation of LRP-6 and IL-6-mediated STAT3 signaling. PloS One. (2013) 8:e85737. doi: 10.1371/journal.pone.0085737 24392029 PMC3877388

[35] Vitale D Kumar Katakam S Greve B Jang B Oh ES Alaniz L. Proteoglycans and glycosaminoglycans as regulators of cancer stem cell function and therapeutic resistance. FEBS J. (2019) 286:2870–82. doi: 10.1111/febs.14967 31230410

[36] Zhou Y Yang D Yang Q Lv X Huang W Zhou Z. Single-cell RNA landscape of intratumoral heterogeneity and immunosuppressive microenvironment in advanced osteosarcoma. Nat Commun. (2020) 11:6322. doi: 10.1038/s41467-020-20059-6 33303760 PMC7730477

[37] Liu Y Feng W Dai Y Bao M Yuan Z He M. Single-cell transcriptomics reveals the complexity of the tumor microenvironment of treatment-naive osteosarcoma. Front Oncol. (2021) 11:709210. doi: 10.3389/fonc.2021.709210 34367994 PMC8335545

[38] Horie T Fukasawa K Yamada T Mizuno S Iezaki T Tokumura K. Erk5 in bone marrow mesenchymal stem cells regulates bone homeostasis by preventing osteogenesis in adulthood. Stem Cells (Dayton Ohio). (2022) 40:411–22. doi: 10.1093/stmcls/sxac011 35304894

[39] Yoshimoto M Sadamori K Tokumura K Tanaka Y Fukasawa K Hinoi E. Bioinformatic analysis reveals potential relationship between chondrocyte senescence and protein glycosylation in osteoarthritis pathogenesis. Front Endocrinol. (2023) 14:1153689. doi: 10.3389/fendo.2023.1153689 PMC10229820 37265706

[40] Tokumura K Sadamori K Yoshimoto M Tomizawa A Tanaka Y Fukasawa K. The bioinformatics identification of potential protein glycosylation genes associated with a glioma stem cell signature. BioMedInformatics. (2024) 4:75–88. doi: 10.3390/biomedinformatics4010005

[41] Wang X Qin G Liang X Wang W Wang Z Liao D. Targeting the CK1α/CBX4 axis for metastasis in osteosarcoma. Nat Commun. (2020) 11:1141. doi: 10.1038/s41467-020-14870-4 32111827 PMC7048933

[42] Fukasawa K Lyu J Kubo T Tanaka Y Suzuki A Horie T. MEK5-ERK5 axis promotes self-renewal and tumorigenicity of glioma stem cells. Cancer Res Commun. (2023) 3:148–59. doi: 10.1158/2767-9764.crc-22-0243 PMC10035453 36968222

[43] Hiraiwa M Fukasawa K Iezaki T Sabit H Horie T Tokumura K. SMURF2 phosphorylation at Thr249 modifies glioma stemness and tumorigenicity by regulating TGF-β receptor stability. Commun Biol. (2022) 5:22. doi: 10.1038/s42003-021-02950-0 35017630 PMC8752672

[44] Fukasawa K Kadota T Horie T Tokumura K Terada R Kitaguchi Y. CDK8 maintains stemness and tumorigenicity of glioma stem cells by regulating the c-MYC pathway. Oncogene. (2021) 40:2803–15. doi: 10.1038/s41388-021-01745-1 33727660

[45] Hinoi E Iezaki T Ozaki K Yoneda Y. Nuclear factor-κB is a common upstream signal for growth differentiation factor-5 expression in brown adipocytes exposed to pro-inflammatory cytokines and palmitate. Biochem Biophys Res Commun. (2014) 452:974–9. doi: 10.1016/j.bbrc.2014.09.022 25223801

[46] Nakamura Y Hinoi E Iezaki T Takada S Hashizume S Takahata Y. Repression of adipogenesis through promotion of Wnt/β-catenin signaling by TIS7 up-regulated in adipocytes under hypoxia. Biochim Biophys Acta. (2013) 1832:1117–28. doi: 10.1016/j.bbadis.2013.03.010 23517917

[47] Yauy K Tran Mau-Them F Willems M Coubes C Blanchet P Herlin C. B3GAT3-related disorder with craniosynostosis and bone fragility due to a unique mutation. Genet medicine: Off J Am Coll Med Genet. (2018) 20:269–74. doi: 10.1038/gim.2017.109 28771243

[48] Gibbs CP Kukekov VG Reith JD Tchigrinova O Suslov ON Scott EW. Stem-like cells in bone sarcomas: implications for tumorigenesis. Neoplasia (New York NY). (2005) 7:967–76. doi: 10.1593/neo.05394 PMC1502023 16331882

[49] Rainusso N Man TK Lau CC Hicks J Shen JJ Yu A. Identification and gene expression profiling of tumor-initiating cells isolated from human osteosarcoma cell lines in an orthotopic mouse model. Cancer Biol Ther. (2011) 12:278–87. doi: 10.4161/cbt.12.4.15951 PMC3173730 21617384

[50] Stevens RL Austen KF. Effect of p-nitrophenyl-beta-D-xyloside on proteoglycan and glycosaminoglycan biosynthesis in rat serosal mast cell cultures. J Biol Chem. (1982) 257:253–9. doi: 10.1016/S0021-9258(19)68354-7 7053369

[51] Kraushaar DC Yamaguchi Y Wang L. Heparan sulfate is required for embryonic stem cells to exit from self-renewal. J Biol Chem. (2010) 285:5907–16. doi: 10.1074/jbc.M109.066837 PMC2820816 20022960

[52] Chen J Sun T You Y Wu B Wang X Wu J. Proteoglycans and glycosaminoglycans in stem cell homeostasis and bone tissue regeneration. Front Cell Dev Biol. (2021) 9:760532. doi: 10.3389/fcell.2021.760532 34917612 PMC8669051

[53] Mizumoto S Ikegawa S Sugahara K. Human genetic disorders caused by mutations in genes encoding biosynthetic enzymes for sulfated glycosaminoglycans. J Biol Chem. (2013) 288:10953–61. doi: 10.1074/jbc.R112.437038 PMC3630846 23457301

[54] Taylan F Mäkitie O. Abnormal proteoglycan synthesis due to gene defects causes skeletal diseases with overlapping phenotypes. Hormone Metab Res = Hormon- und Stoffwechselforschung = Hormones metabolisme. (2016) 48:745–54. doi: 10.1055/s-0042-118706 27871115

[55] Kang HG Evers MR Xia G Baenziger JU Schachner M. Molecular cloning and characterization of chondroitin-4-O-sulfotransferase-3. A novel member of the HNK-1 family of sulfotransferases. J Biol Chem. (2002) 277:34766–72. doi: 10.1074/jbc.M204907200 12080076

[56] Nassar D Blanpain C. Cancer stem cells: basic concepts and therapeutic implications. Annu Rev Pathol. (2016) 11:47–76. doi: 10.1146/annurev-pathol-012615-044438 27193450

[57] Lindsey BA Markel JE Kleinerman ES. Osteosarcoma overview. Rheumatol Ther. (2017) 4:25–43. doi: 10.1007/s40744-016-0050-2 27933467 PMC5443719

[58] Toguchida J Ishizaki K Sasaki MS Nakamura Y Ikenaga M Kato M. Preferential mutation of paternally derived RB gene as the initial event in sporadic osteosarcoma. Nature. (1989) 338:156–8. doi: 10.1038/338156a0 2918936

[59] Wunder JS Gokgoz N Parkes R Bull SB Eskandarian S Davis AM. TP53 mutations and outcome in osteosarcoma: a prospective, multicenter study. J Clin oncology: Off J Am Soc Clin Oncol. (2005) 23:1483–90. doi: 10.1200/jco.2005.04.074 15735124

[60] Stephens PJ Greenman CD Fu B Yang F Bignell GR Mudie LJ. Massive genomic rearrangement acquired in a single catastrophic event during cancer development. Cell. (2011) 144:27–40. doi: 10.1016/j.cell.2010.11.055 21215367 PMC3065307

[61] Savage SA Mirabello L Wang Z Gastier-Foster JM Gorlick R Khanna C. Genome-wide association study identifies two susceptibility loci for osteosarcoma. Nat Genet. (2013) 45:799–803. doi: 10.1038/ng.2645 23727862 PMC3910497

[62] Chen X Bahrami A Pappo A Easton J Dalton J Hedlund E. Recurrent somatic structural variations contribute to tumorigenesis in pediatric osteosarcoma. Cell Rep. (2014) 7:104–12. doi: 10.1016/j.celrep.2014.03.003 PMC4096827 24703847

