# Supplementary material for: Role of proteoglycan synthesis genes in osteosarcoma stem cells
Source: Front Oncol. 2024 Apr 16;14:1325794. doi: 10.3389/fonc.2024.1325794 (PMC11058990; doi:10.3389/fonc.2024.1325794)
Supplement: Supplementary file 1 [file DataSheet_1.docx]

Supplementary Material

Role of Proteoglycan Synthesis Genes in Osteosarcoma Stem Cells

Ryoma Osumi^1,†^, Kengo Sugihara^1,†^, Makoto Yoshimoto^1^, Kazuya Tokumura^1^, Yuki Tanaka^1^, and Eiichi Hinoi^1,2,3^*

^1^Department of Bioactive Molecules, Pharmacology, Gifu Pharmaceutical University, Gifu, Japan

^2^United Graduate School of Drug Discovery and Medical Information Sciences, Gifu University, Gifu, Japan

^3^Center for One Medicine Innovative Translational Research, Division of Innovative Modality Development, Gifu University, Gifu, Japan

***Correspondence:**

Eiichi Hinoi

[hinoi-e@gifu-pu.ac.jp](mailto:hinoi-e@gifu-pu.ac.jp)

^†^These authors contributed equally to this work and share first authorship

Keywords: Osteosarcoma, Osteosarcoma stem cell, Proteoglycan, Glycosaminoglycan, *β-1,3-glucuronyltransferase 3*

**Supplementary Figure 1. (A)** t-SNE plot of cell clusters classified in OS tissues (GSE162454). **(B)** Violin plots showing the normalized expression levels of 27 representative marker genes across 11 clusters. **(C)** Barplot showing the expression levels of PG/GAG genes between OSCs and non-OSCs. *SOX2*, NES, and *MYC* co-expressing cells were defined as OSCs (**P* < 0.05, ***P* < 0.01, ****P* < 0.001).

**Supplementary Figure 2.** **(A)** Representative image of 143B tumorspheres (scale bar = 50 μm). **(B)** The frequency of stem cells was determined using Extreme Limiting Dilution Analysis (ELDA) software. **(C)** The proportion of CD133^+^ cells cultured under adherent (left) or floating (right) conditions were determined by flow cytometry. **(D)** The differentiation potential of 143B tumorspheres was observed through morphological changes over time in adherent culture condition. Representative images of 143B tumorspheres at 3 hours (left) and at 19 hours (right) after initiating adherent culture (scale bar = 50 μm). **(E)** Tumor formation in nude mice following transplantation of 143B cells. Representative images of a tumor-bearing nude mouse (left) and the tumor (right).

**Supplementary Table.** RT-qPCR primers list.

| Gene | Forward (5’-3’) | Reverse (5’-3’) |
| --- | --- | --- |
| *ABCG1* | AAGGATTGAATGCAGGTTCCA | AGAGTTGGCCAATGTCTTCTCAC |
| *BMI1* | GTGTTAGGAAAGATGGGAAATGGT | AGCGTAATAACAAGAACGATCAAGG |
| *B3GAT3* | GCAGTCTTCTGAGCCACCTTG | GCTTCATCTTGGGCTTCTCTGT |
| *GAPDH* | AAATCCCATCACCATCTTCCA | AATGAGCCCCAGCCTTCTC |
| *KLF4* | GATGCTCACCCCACCTTCTT | CTTCCCCTCTTTGGCTTGG |
| *SOX2* | AGAGGAGAGTAAGAAACAGCATGGA | GTGAGTGTGGATGGGATTGGT |
